# In Situ Growth of Metal Sulfide Nanocrystals in Poly(3-hexylthiophene): [6,6]-Phenyl C61-Butyric Acid Methyl Ester Films for Inverted Hybrid Solar Cells with Enhanced Photocurrent

**DOI:** 10.1186/s11671-018-2596-0

**Published:** 2018-06-20

**Authors:** Chunyan Yang, Yingying Sun, Xinjie Li, Cheng Li, Junfeng Tong, Jianfeng Li, Peng Zhang, Yangjun Xia

**Affiliations:** 1 Key Lab of Optoelectronic Technology and Intelligent Control of Education Ministry, Lanzhou Jiaotong University, Lanzhou, 730000 China; 2 College of Chemical and Biological Engineering, Lanzhou Jiaotong University, Lanzhou, 730000 China

**Keywords:** Hybrid solar cells (HSCs), P3HT:PC_61_BM, CdS or Sb_2_S_3_ nanocrystals, In situ, Cadmium or antimony xanthate precursor

## Abstract

It has been reported that the performance of bulk heterojunction organic solar cells can be improved by incorporation of nano-heterostructures of metals, semiconductors, and dielectric materials in the active layer. In this manuscript, CdS or Sb_2_S_3_ nanocrystals were in situ generated inside the poly(3-hexylthiophene): [6,6]-phenyl C61-butyric acid (P3HT:PC_61_BM) system by randomly mixing P3HT and PC_61_BM in the presence of cadmium or antimony xanthate precursor. Hybrid solar cells (HSCs) with the configurations of tin-doped indium oxide substrate (ITO)/CdS interface layer/P3HT:PC_61_BM: x wt.% CdS/MoO_3_/Ag and ITO/CdS interface layer /P3HT:PC_61_BM: x wt.% Sb_2_S_3_/MoO_3_/Ag were fabricated. Hybrid active layers (P3HT:PC_61_BM: x wt.% CdS or P3HT:PC_61_BM: x wt.% Sb_2_S_3_) were formed completely by thermally annealing the film resulting in the decomposition of the cadmium or antimony xanthate precursor to CdS or Sb_2_S_3_ nanocrystals, respectively. The effects of x wt.% CdS (or Sb_2_S_3_) nanocrystals on the performance of the HSCs were studied. From UV–Vis absorption, hole mobilities, and surface morphological characterizations, it has been proved that incorporation of 3 wt.% CdS (or Sb_2_S_3_) nanocrystals in the active layer of P3HT:PC_61_BM-based solar cells improved the optical absorption, the hole mobility, and surface roughness in comparison with P3HT:PC_61_BM-based solar cells, thus resulting in the improved power conversion efficiencies (PCEs) of the devices.

**Electronic supplementary material:**

The online version of this article (10.1186/s11671-018-2596-0) contains supplementary material, which is available to authorized users.

## Background

Organic semiconductors-based photovoltaic devices are complimented by various advantageous characteristics, such as light weight, low cost, ease of solution-based roll-to-roll large-scale manufacture and compatibility with flexible materials [1, 2]. Besides, inorganic semiconductors are ideal for highly efficient solar cells for their superior charge mobility, chemical stability, as well as the enhanced light absorption (potential to tune their optical band gap into the near-infrared region) [3, 4]. Therefore, hybrid solar cells (HSCs), composed of both organic semiconductors and inorganic semiconductors, have garnered considerable attention mainly due to the promise of integrating the attractive qualities of both classes of materials [5–7]. A typical HSC is based on the bulk heterojunction concept in which a blend of organic materials and inorganic nanoparticles active layer sandwiched between two charge collecting electrodes [5–7]. To date, a wide range of organic materials, such as low band gap conjugated polymers [7], along with many inorganic materials, including metal nanomaterials (Ag, Au) [8, 9], silicon [10, 11], metal oxide nanoparticles (ZnO, TiO_2_) [12–14], silicon dioxide nanoparticles (SiO_2_) [15], cadmium compounds (CdS, CdSe, CdTe) [16–18], low band gap nanoparticles (PbS, PbSe, Sb_2_S_3_,Cu_2_S, SnS_2_, CuInS_2_, FeS_2_) [19–25], and so on, have been applied as the active layer in HSCs.

The performance of HSCs depends critically on the ability to control materials and interface structure at the nanometer length scale [26]. The conventional method for preparing the organic and inorganic composite active layer is directly mixing a given organic polymer with the as-prepared ligand-capped inorganic nanocrystal by using a cosolvent [16–25]. Although incorporation of the surface ligands into the organic/inorganic composite can improve distribution of nanocrystals in a conjugated polymer, the long chain ligands would form an insulating interface between the polymer matrix and the nanocrystals. To be a result, the insulating interface will inhibit charge transfer between the polymer and the nanocrystals, and the cosolvent will adversely affect polymer chain orientation as well as the solubility of inorganic nanocrystals, thereby affect the power conversion efficiencies (PCEs) of the devices [5, 27]. These drawbacks can largely be circumvented by several alternative strategies, including ligand exchange [27, 28], the use of thiols, poly- and oligothiophenes, and amine-functionalized copolymer surfactants [29–31], employing of thermally cleavable ligands [32] and the synthesis of nanocrystals in conducting polymer solution [33]. Another alternative strategy is in situ generation of the inorganic semiconductor inside the organic material without surfactants or ligands [26, 34, 35]. In the process, a polymer solution containing a well-soluble organometallic precursor of the inorganic nanoparticles is deposited. Because the organometallic precursor is readily soluble in organic media, it can be cast into a thin film together with the polymer from solution. Upon thermal decomposition of the film, the organometallic precursor is converted into inorganic material with the polymer layer, ensuring intimate mixing and concomitantly efficient photo-induced charge transport. In this way, the hybrid active layers can be formed under the condition of no surface ligands and cosolvent. Haque’s group has reported a general method based upon the controlled in situ thermal decomposition of a single-source metal xanthate precursor inside a polymer film [26, 36–40]. Photovoltaic devices based upon such hybrid layers with the configuration of tin-doped indium oxide substrate (ITO)/TiO_2_/CdS interface layer/P3HT: CdS/poly(3,4-ethylenedioxythiophene) doped with polystyrene sulfonic acid (PEDOT:PSS)/Ag and ITO/TiO_2_/CdS interface layer/P3HT: Sb_2_S_3_/PEDOT:PSS/Ag were shown to exhibit PCEs of 2.17 and 1.29%, respectively [36, 38].

In this article, for the considerations of using the synergistic effects between P3HT:PC_61_BM-based solar cells and metal sulfide nanocrystals (CdS and Sb_2_S_3_) as a doped material for offering superior charge mobility and enhancing light absorption, we report the HSCs with the configuration of ITO/CdS interface layer/P3HT: PC_61_BM: x wt.% CdS/MoO_3_/Ag and ITO/CdS interface layer/P3HT:PC_61_BM: x wt.% Sb_2_S_3_/MoO_3_/Ag. Here, ITO and Ag were made as cathode and the top anode, while CdS interface layer and MoO_3_ were used for electron and hole transporting layers, respectively. CdS or Sb_2_S_3_ nanocrystals were in situ generated inside the P3HT:PC_61_BM system by randomly mixing P3HT and PC_61_BM in the presence or absence of cadmium or antimony xanthate precursor. Hybrid active layers (P3HT:PC_61_BM: x wt.% CdS or P3HT:PC_61_BM: x wt.% Sb_2_S_3_) were formed completely by thermally annealing the film resulting in the decomposition of the cadmium or antimony xanthate precursor to CdS or Sb_2_S_3_ nanocrystals, respectively. The effects of x wt.% CdS (or Sb_2_S_3_) nanocrystals on the performance of P3HT:PC_61_BM-based HSCs were studied. And the highest PCEs of 2.91 and 2.92% were obtained for the HSCs with 3 wt.% CdS nanocrystals and 3 wt.% Sb_2_S_3_ nanocrystals, respectively. UV–Vis absorption, hole mobilities, and surface morphological characterizations of the active layers have been carried out in order to understand the probable reasons for the improvement of the device performance.

## Methods/Experimental

### Fabrication and Characterization of HSCs

The organic/inorganic HSCs, with device configuration of ITO/CdS interface layer /P3HT:PC_61_BM:x wt.% CdS or Sb_2_S_3_/MoO_3_/Ag were fabricated as follows: firstly, cadmium xanthate precursor (Di(ethylxanthato-*κ*^2^*S*,*S*′)bis(pyridine-κ *N*)cadmium(II), Cd(S_2_COEt)_2_(C_5_H_4_N)_2_, Et = ethy) and antimony xanthate precursor (Tri(ethylxanthato-*κ*^2^*S*,*S*′)antimony(III), Sb(S_2_COEt)_3_) were prepared respectively following the previously published procedure [26, 38, 39]. Secondly, a patterned ITO-coated glass with a sheet resistance of 10~15 Ω square^−1^ was cleaned in de-ionized water, acetone, and isopropanol in turn. After that, CdS interface layer (10 nm) was deposited as electron transporting layer following the previously published work [41], from a 100 mg/mL chlorobenzene solution of Cd(S_2_COEt)_2_(C_5_H_4_N)_2_ by spin coating at 6000 rpm for 40 s followed by annealing at 160 °C for 15 min in a nitrogen glove box. The active layer was deposited on top of the CdS interface layer. The pristine P3HT:PC_61_BM at 1:1 weight-ratio solution in chlorobenzene with the concentration of 17 mg mL^−1^ of P3HT was prepared. To form the hybrid solution, cadmium xanthate precursor (Cd(S_2_COEt)_2_(C_5_H_4_N)_2_) or antimony xanthate precursor (Sb(S_2_COEt)_3_) were added to the pristine solution (x wt.% CdS or Sb_2_S_3_ with respect to the weight of P3HT). The active layer was spin-casting from these blend solutions at 600 rpm for 40 s followed by annealing on a hot plate at 160 °C for 30 min in a glove box. In a control experiment, P3HT:PC_61_BM-only layer (at 1:1 weight-ratio solution in chlorobenzene with the concentration of 17 mg mL^−1^ of P3HT) without Cd(S_2_COEt)_2_(C_5_H_4_N)_2_ or Sb(S_2_COEt)_3_ was also spin-coated and annealed at the same experimental conditions. Then, the samples were transferred into a high vacuum chamber (under vacuum of 3 × 10^−5^ Pa) to complete the HSCs, where an 8-nm-thick MoO_3_ hole collecting layer and a 100-nm-thick Ag anode were thermally evaporated through shadow masks. The thickness of the evaporated cathode was monitored by a quartz crystal thickness/ratio monitor (SI-TM206, Shenyang Science Co.). In addition, each device had an active area of 0.10 cm^2^. All the fabrication processes were carried out inside a controlled atmosphere in a nitrogen drybox (Etelux Co.) containing less than 1 ppm oxygen and moisture.

### Thin Film and Device Characterization

X-ray diffraction (XRD) data were measured on a PAN alytical X’Pert Pro X-ray diffractometer equipped with graphite monochromatized Cu Kα radiation (λ = 1.541874 Å). The accelerating voltage was set at 40 kV with 40 mA flux in the 2θ range of 10–70°.Thermogravimetric analysis (TGA) measurements of metal xanthate precursor complex were performed on a thermal analysis system (pyris diamond 6300, PerkinElmer) under a heating rate of 10 °C min^−1^ and a nitrogen flow rate of 20 mL min^−1^. UV–Vis absorption measurements of the samples were recorded at room temperature with a U-3900H spectrophotometer (Shanghai Tianmei). The PCEs of the resulting HSCs were measured under 1 sun, AM 1.5G (Air mass 1.5 global) condition using a solar simulator (XES-70S1, San-EI Electric Co.) (100 mW cm^−2^). The current density–voltage (J–V) characteristics were recorded with a Keithley 2410 source measurement unit in the nitrogen drybox (Etelux Co.). The spectral responses of the devices were measured with a commercial EQE/incident photon to current conversion efficiency (IPCE) setup (7-SCSpecIII, Beijing 7-star Optical Instruments Co., Ltd.). A calibrated silicon detector was used to determine the absolute photosensitivity. Tapping-mode atomic force microscopy (AFM) images were obtained using a MFP-3D-SA system (Asylum Research).

## Results and Discussion

**Fig. 1:**
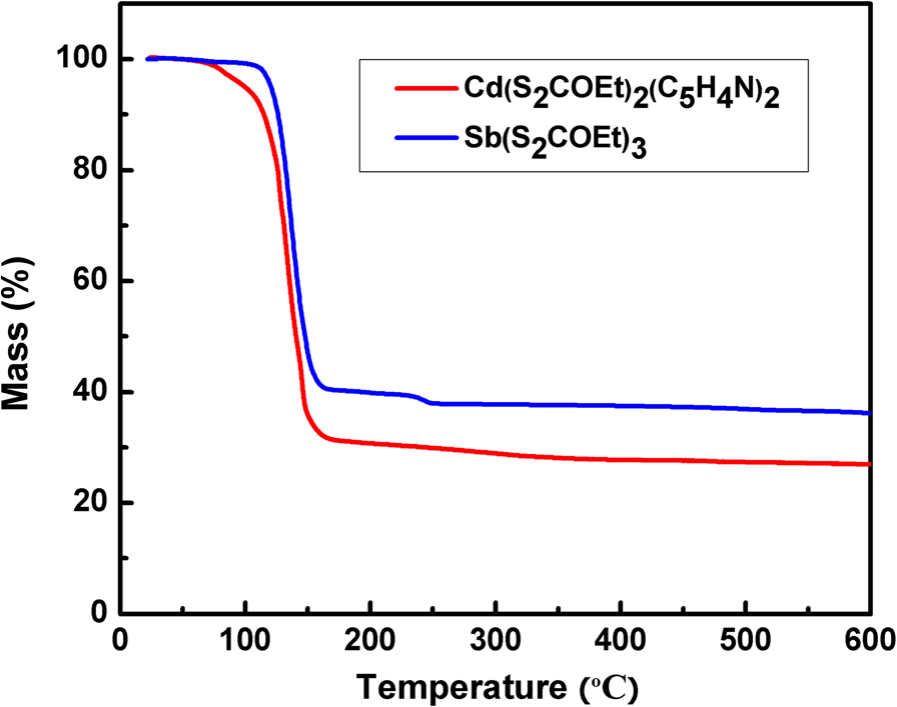
TGA plots of Cd(S_2_COEt)_2_(C_5_H_4_N)_2_ and Sb(S_2_COEt)_3_

Thermal stabilities of Cd(S_2_COEt)_2_(C_5_H_4_N)_2_ and Sb(S_2_COEt)_3_ were investigated by TGA firstly, as shown in Fig. 1. Cd(S_2_COEt)_2_(C_5_H_4_N)_2_ begins decomposing at about 50 °C and is complete by 150 °C, the final residual mass (about 25.0%) is close to that of CdS (28.1%), which was also proved in the previous work [41]. Sb(S_2_COEt)_3_ begins decomposing at about 120 °C and is complete by 160 °C, and the weight remaining (35.8%) corresponds to Sb_2_S_3_ (35.0%), which is consistent with the earlier work [42].

**Fig. 2:**
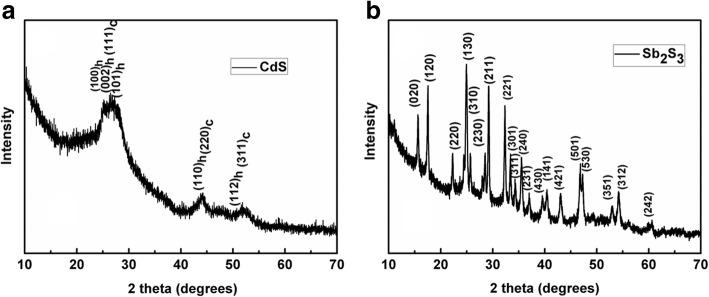
XRD patterns of the thin films obtained by thermal decomposition of **a** (Cd(S_2_COEt)_2_(C_5_H_4_N)_2_) and **b** Sb(S_2_COEt)_3_, respectively

Thin films were spin coated from chlorobenzene solution of Cd(S_2_COEt)_2_(C_5_H_4_N)_2_ or Sb(S_2_COEt)_3_ firstly, then were annealed at 160 °C for 30 min. As a result, the yellow or orange thin films were obtained, respectively. In order to characterize the structure properties of the thin films, XRD studies of the annealed films were performed. The XRD patterns of the product are shown in Fig. 2. According to the reference patterns for hexagonal CdS (PDF 41–1049) and cubic CdS (PDF 01–080–0019), it is apparent that the diffraction peaks in Fig. 2a can be indexed to a blend of hexagonal and cubic crystal structure, which were shown above the peaks (h and c indicate the hexagonal and cubic phase, respectively), as described in the previous article [37]. The diffraction peaks in Fig. 2b can be fully indexed to the orthorhombic phase of Sb_2_S_3_ (cell constants *a* = 11.23 Å, *b* = 11.31 Å, *c* = 3.841 Å; JCPDS card file 42–1393) [43, 44], which is in good agreement with the TG results in Fig. 1.

The surface morphologies of CdS and Sb_2_S_3_ thin films have also been exploited. Additional file 1: Figure S1 presents the morphology evolution of ITO before (Additional file 1: Figure S1a) and after thermal decomposition (160 °C, 15 min) of the chlorobenzene solution of Cd(S_2_COEt)_2_(C_5_H_4_N)_2_ (Additional file 1: Figure S1b) and Sb(S_2_COEt)_3_ (Additional file 1: Figure S1c). As described in our previous work [41], it can be seen that the surface of bare ITO shows a densely packed gathering of fine crystals with the grain size of about 10 nm. After thermal decomposition of the chlorobenzene solution of cadmium or antimony xanthate precursor, it is apparent that CdS nanocrystal (about 60~100 nm) film or Sb_2_S_3_ nanocrystal (100~200 nm sized clusters) film is formed on ITO substrate.

**Fig. 3:**
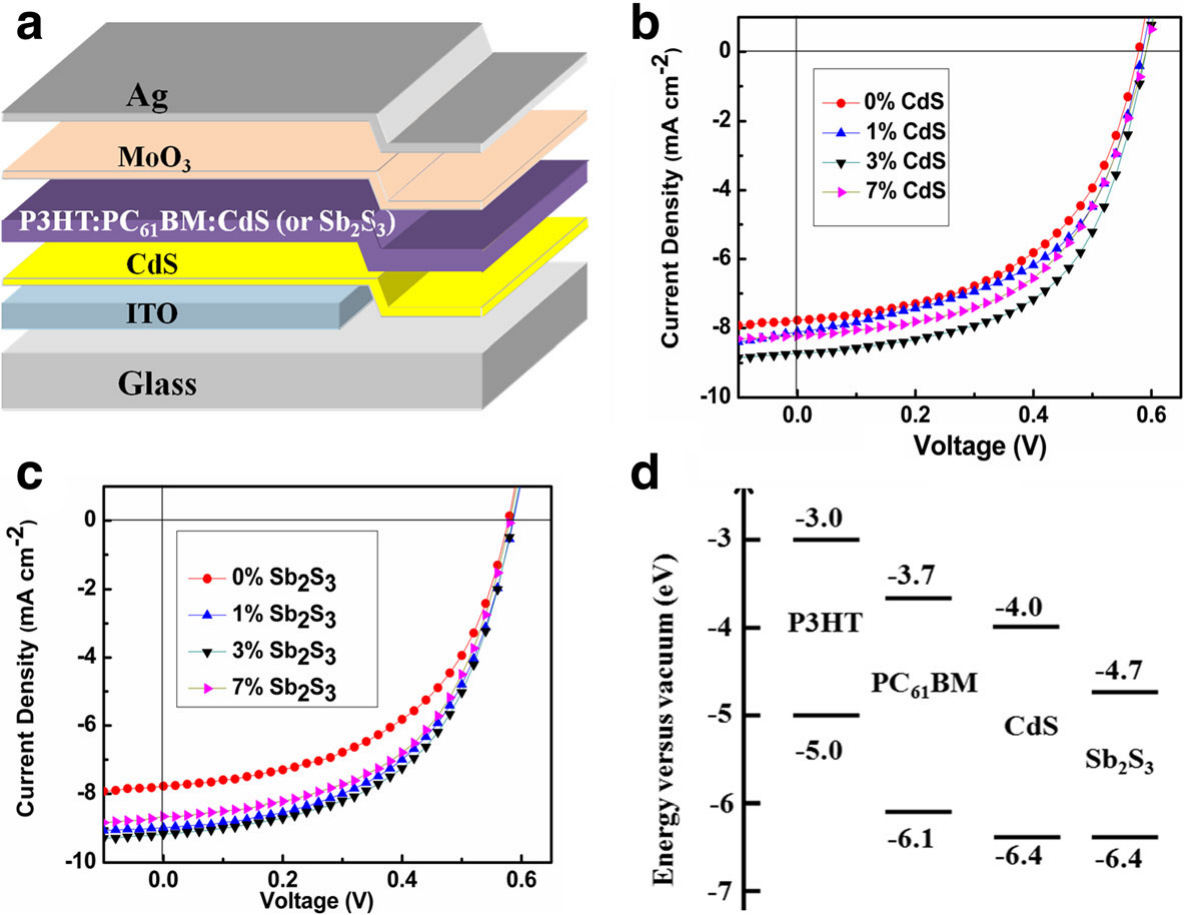
**a** Schematic diagram of the HSCs. **b** J–V curves of the HSCs based on P3HT:PC_61_BM: CdS with different wt.% of CdS nanocrystals. **c** J–V curves of the HSCs based on P3HT:PC_61_BM: Sb_2_S_3_ with different wt.% of Sb_2_S_3_ nanocrystals. **d** Energy band diagram of the materials used in the active layer in the HSCs

In order to study the effect of CdS (or Sb_2_S_3_) nanocrystals on the performance of P3HT:PC_61_BM-based HSCs, devices have been fabricated using the structure ITO/CdS interface layer/P3HT:PC_61_BM: x wt.% CdS (or Sb_2_S_3_)/MoO_3_/Ag as shown in Fig. 3a. CdS or Sb_2_S_3_ nanocrystals were in situ generated inside the P3HT:PC_61_BM system by randomly mixing P3HT and PC_61_BM in the presence or absence of cadmium or antimony xanthate precursor. Hybrid active layers (P3HT:PC_61_BM: x wt.% CdS or P3HT:PC_61_BM: x wt.% Sb_2_S_3_) were formed completely by thermally annealing the film causing the cadmium or antimony xanthate precursor decomposited into CdS or Sb_2_S_3_ nanocrystals, respectively (SEM images of P3HT:PC_61_BM, P3HT:PC_61_BM:3 wt.% CdS, and P3HT:PC_61_BM:3 wt.% Sb_2_S_3_ films on ITO substrates were shown in Additional file 1: Figure S2). Annealing temperature of 160 °C and annealing time for 30 min were chosen in our experiment in order to make the cadmium or antimony xanthate precursor decompose completely (see the TGA plots of Cd(S_2_COEt)_2_(C_5_H_4_N)_2_ and Sb(S_2_COEt)_3_ in Fig. 1).

J–V characteristics of the HSCs with different wt.% CdS (or Sb_2_S_3_) nanocrystals incorporated into the organic layers are shown in Fig. 3b (or Fig. 3c), and the photovoltaic parameters, including short-circuit current (*J*_sc_), open-circuit voltage (*V*_oc_), fill factor (FF), the series resistance (*R*_S_), and PCE, are listed in Table 1. All given data are the average values calculated from more than 20 devices. The device without CdS (or Sb_2_S_3_) nanocrystals (ITO/CdS interface layer/P3HT:PC_61_BM: /MoO_3_/Ag) showed a *J*_sc_ of 7.77 mAcm^−2^, a *V*_oc_ of 0.58 V, a FF of 0.52, and a PCE of 2.34%. With addition of CdS or Sb_2_S_3_ nanocrystals, it was observed that *V*_oc_, which is limited by the energy difference between the highest occupied molecular orbital (HOMO) level of polymer donor and lowest unoccupied molecular orbital (LUMO) level of the acceptor in polymer solar cells [45, 46], remains around 0.58–0.59 V in all HSCs. This can be understood from the fact that PC_61_BM is acting as the acceptor and CdS or Sb_2_S_3_ might be acting as an electron cascade (the energy band diagram of the materials used in the active layer in the HSCs is shown in Fig. 3d), as mentioned in the previous work [47]. For the HSCs with addition of CdS, *J*_sc_ firstly increases from 7.77 to 8.72 mA cm^−2^ with the increase of CdS from 0 to 3 wt.%, and then decreases to 8.23 mA cm^−2^ when the mass of CdS further increases from 3 to 7 wt.%. Simultaneously, *R*_S_ reduces obviously from 22.15 Ω cm^2^ (0 wt.% CdS) to 16.70 Ω cm^2^ (3 wt.% CdS), contributing to a remarkable increase of FF from 0.52 to 0.56. As a result, the HSC with 3 wt.% CdS nanocrystals yields the best device performance, providing the PCE of 2.91%. It is worth mentioning here that this value is much higher than the best PCE of 0.95% which Chand’s group has been obtained in the HSCs (ITO/PEDOT:PSS/P3HT:PC_61_BM:CdS/Al) using CdS nanocrystals fabricated by solution chemistry as one of the components in active layer [48]. The change rules of *J*_sc_ and FF in the HSCs with addition of Sb_2_S_3_ were similar to that in the HSCs with addition of CdS, except for the more obvious increase of *J*_sc_ (from 7.77 to 9.15 mA cm^−2^) with the increase of Sb_2_S_3_ from 0 to 3 wt.%. Coincidentally, the device with 3 wt.% Sb_2_S_3_ nanocrystals also provides the highest PCE of 2.92% with *J*_sc_ of 9.15 mAcm^−2^, *V*_oc_ of 0.58 V, FF of 0.54.

**Table 1:** Photovoltaic properties of the HSCs based on P3HT:PC_61_BM: CdS or P3HT:PC_61_BM: Sb_2_S_3_ with different wt.% of CdS or Sb_2_S_3_ nanocrystals

Photoactive blend	*V*_oc_ (*V*)	*J*_sc_ (mA cm^−2^)	FF	PCE (%)	*0*s (Ω cm^2^)
0% CdS or Sb_2_S_3_	0.58	7.77	0.52	2.34	22.15
1% CdS	0.58	8.12	0.53	2.51	19.40
3% CdS	0.59	8.72	0.56	2.91	16.70
7% CdS	0.59	8.23	0.54	2.63	20.74
1% Sb_2_S_3_	0.58	8.97	0.53	2.80	19.14
3% Sb_2_S_3_	0.58	9.15	0.54	2.92	17.98
7% Sb_2_S_3_	0.58	8.65	0.54	2.73	18.70

**Fig. 4:**
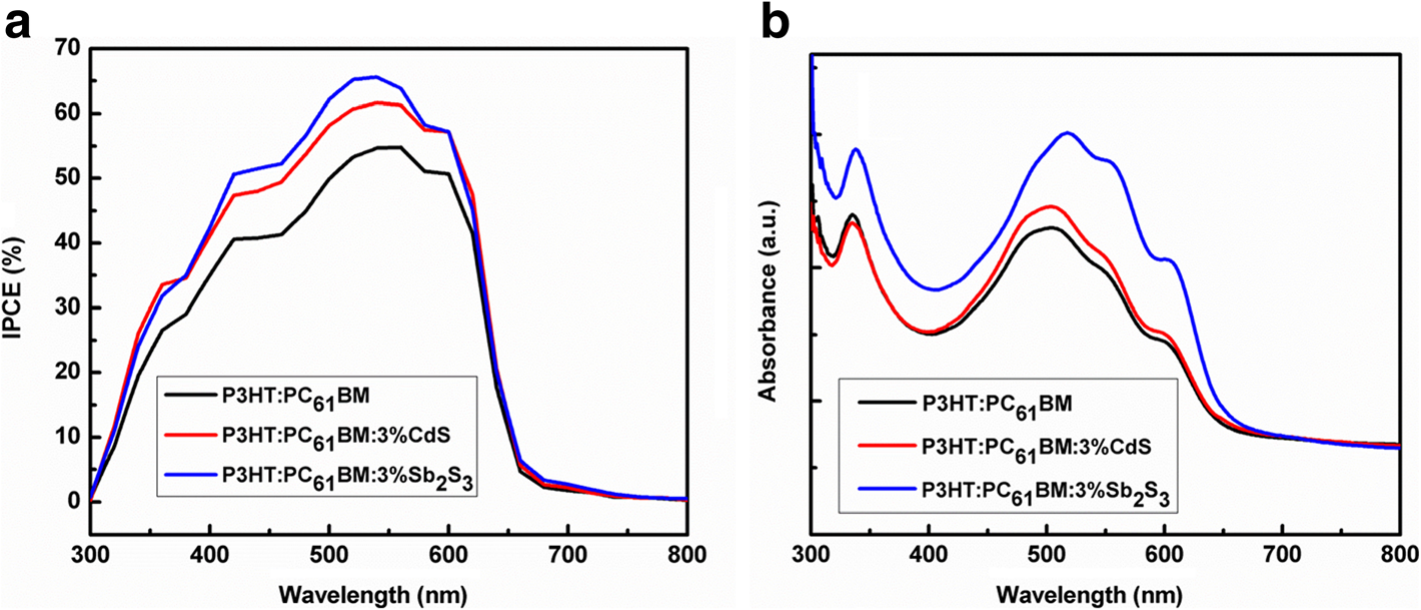
**a** IPCE curves of the HSCs based on P3HT:PC_61_BM, P3HT:PC_61_BM:3 wt.% CdS, and P3HT:PC_61_BM:3 wt.% Sb_2_S_3_. **b** UV–Vis absorbance spectra of the films of P3HT:PC_61_BM, P3HT:PC_61_BM:3 wt.% CdS, and P3HT:PC_61_BM:3 wt.% Sb_2_S_3_

Another useful parameter for determining the PCE of the HSCs is the IPCE, which reaches 100% when all incident photons generate electron hole pairs. However, in practical situations, because of losses caused by the reflection of incident photons, imperfect absorption of photons by the semiconductor, and recombination of charge carriers within the semiconductor, IPCE is typically less than 100% [8]. IPCE spectra for the photovoltaic devices based on P3HT:PC_61_BM, P3HT:PC_61_BM: 3 wt.% CdS, and P3HT: PC_61_BM: 3 wt.% Sb_2_S_3_ are displayed in Fig. 4a for comparison. Although all IPCE spectra are similar in shape, the IPCE value for the HSCs containing P3HT:PC_61_BM: 3 wt.% CdS (or P3HT: PC_61_BM: 3 wt.% Sb_2_S_3_) is higher than that for the P3HT:PC_61_BM in all wavelength (300–650 nm). For example, the photovoltaic device of P3HT:PC_61_BM was found to have an IPCE maximum near 55% at 540 nm and the IPCEs of the HSCs with P3HT:PC_61_BM: 3 wt.% CdS and P3HT:PC_61_BM: 3 wt.% Sb_2_S_3_ are 60 and 65% at the same wavelength, respectively.

In order to understand the probable reasons for improvement in the device performance by addition of CdS or Sb_2_S_3_ nanocrystals, UV–Vis absorption studies firstly have been carried out on the films of P3HT:PC_61_BM, P3HT:PC_61_BM:3 wt.% CdS, and P3HT:PC_61_BM:3 wt.% Sb_2_S_3_ with the same thickness. UV-Visible absorption spectra in Fig. 4b show that the absorption of the films addition of CdS or Sb_2_S_3_ nanocrystals in P3HT:PC_61_BM were almost similar with that of P3HT:PC_61_BM, while absorption of P3HT:PC_61_BM: 3 wt.% CdS was slightly higher than that of P3HT:PC_61_BM. Furthermore, the absorption of P3HT:PC_61_BM: 3 wt.% Sb_2_S_3_ was obviously higher than that of P3HT:PC_61_BM. That is, the embedment of 3 wt.% CdS or Sb_2_S_3_ in P3HT:PC_61_BM matrix properly improved the optical absorption in comparison with P3HT:PC_61_BM, therefore improving the *J*_SC_ of the devices.

**Fig. 5:**
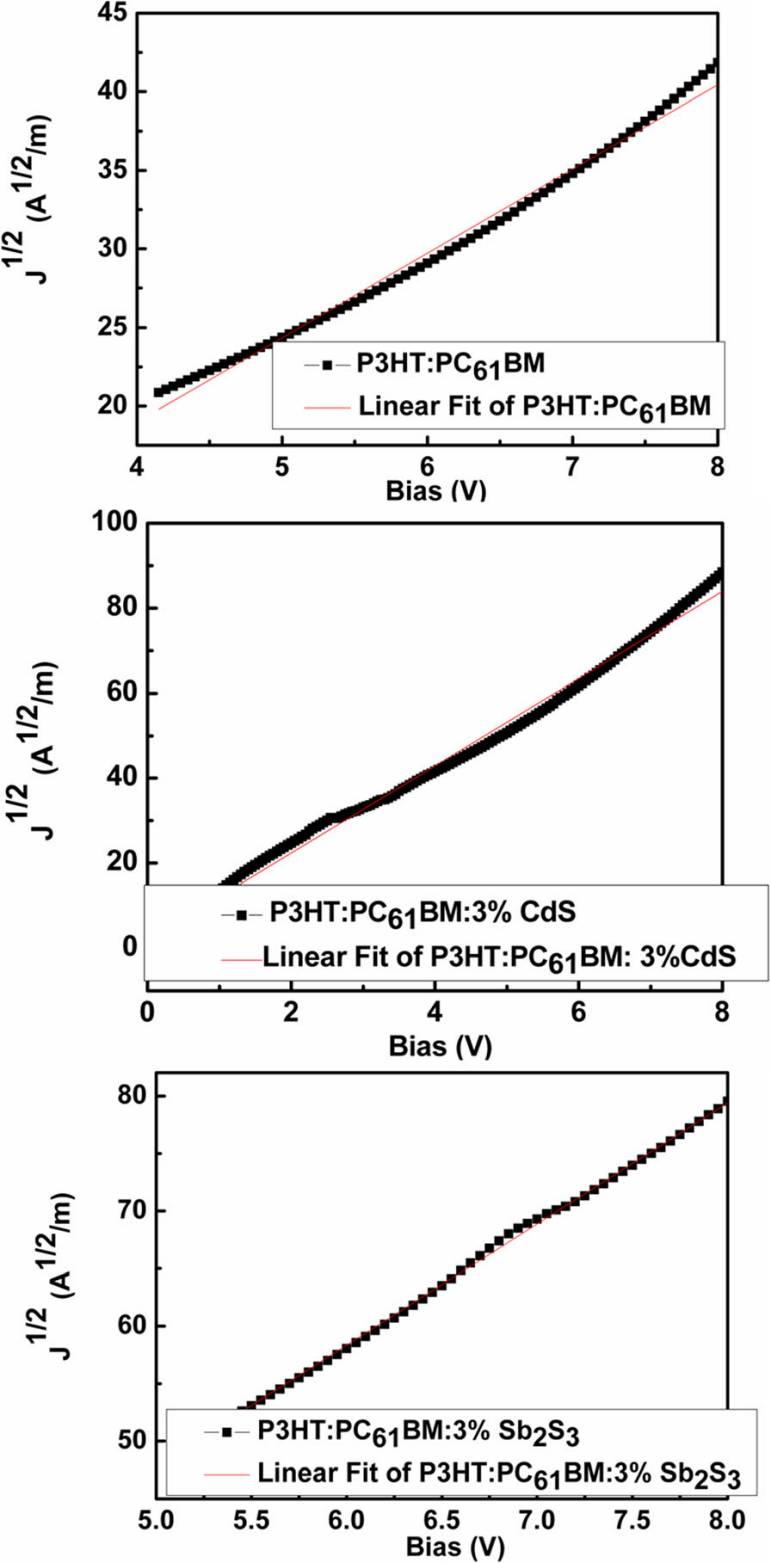
J^1/2^–V curves of the hole-only devices for the devices with P3HT:PC_61_BM, P3HT:PC_61_BM:3 wt.% CdS and P3HT:PC_61_BM:3 wt.% Sb_2_S_3_ as the active layer, respectively

After that, the hole mobilities of devices based on P3HT:PC_61_BM, P3HT:PC_61_BM:3 wt.% CdS, and P3HT:PC_61_BM:3 wt.% Sb_2_S_3_ were determined by applying the space-charge limited current (SCLC) model [49]. Figure 5 shows J^1/2^–V curves of the hole-only devices (ITO/PEDOT:PSS/P3HT:PC_61_BM(or P3HT:PC_61_BM:3 wt.% CdS or P3HT:PC_61_BM:3 wt.% Sb_2_S_3_)/MoO_3_/Ag). The apparent hole mobilities calculated from SCLC model were found to be 4.09 × 10^−5^ cm^2^ V^−1^ s^−1^, 1.53 × 10^−4^ cm^2^ V^−1^ s^−1^, and 1.69 × 10^−4^ cm^2^ V^−1^ s^−1^ for the devices with P3HT:PC_61_BM, P3HT:PC_61_BM:3 wt.% CdS, and P3HT:PC_61_BM:3 wt.% Sb_2_S_3_ as the active layer, respectively. Obviously, the hole mobility increases when 3 wt.% CdS or Sb_2_S_3_ embedded in P3HT:PC_61_BM matrix. Studies have shown that in P3HT:PC_61_BM, electron mobility is higher than hole mobility and this carrier imbalance like that is detrimental to photovoltaic performance [9, 50]. The increase in hole mobility of device based on P3HT:PC_61_BM:3 wt.% CdS or P3HT:PC_61_BM:3 wt.% Sb_2_S_3_ allows more balanced charge transport in the active layer, thus improving the *J*_SC_ and FF, furthermore improving the PCE of the device, as mentioned in the previous work [9].

**Fig. 6:**
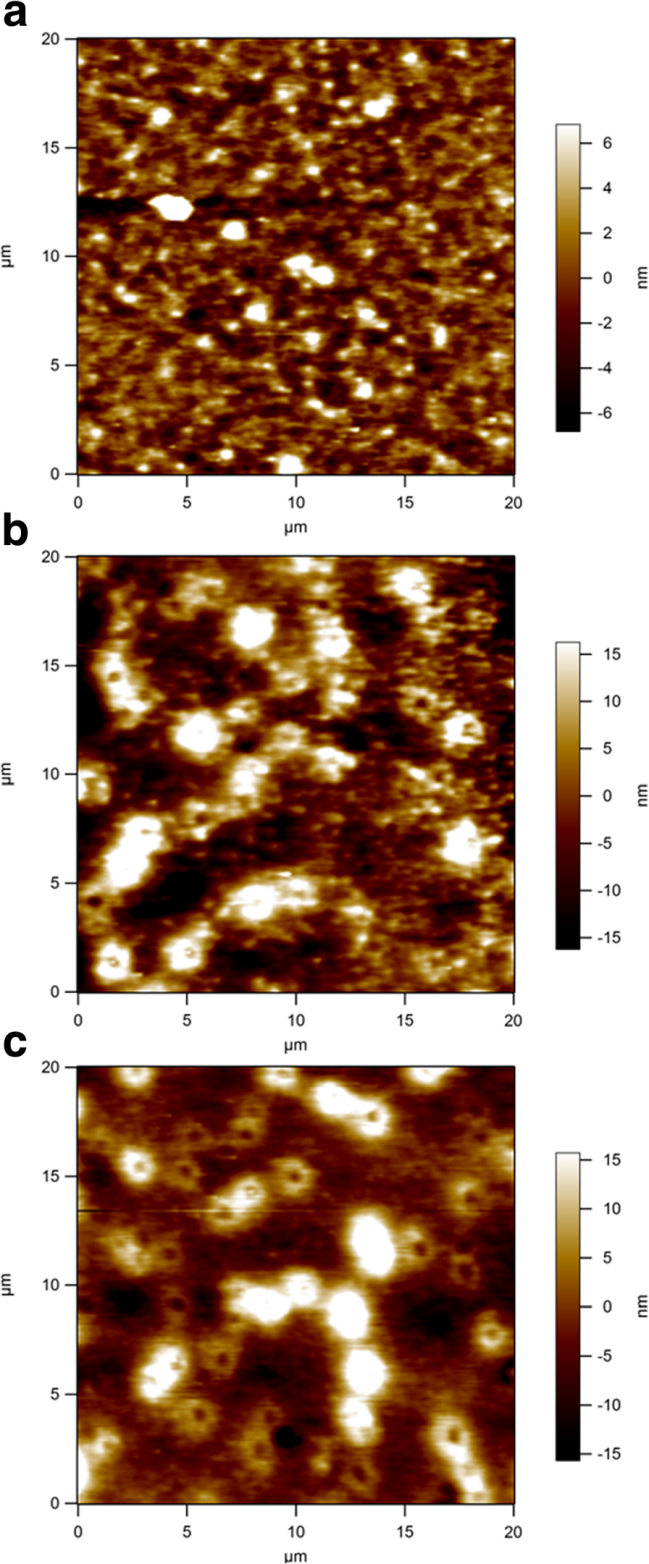
AFM height images of **a** P3HT:PC_61_BM, **b** P3HT:PC_61_BM:3 wt.% CdS, and **c** P3HT:PC_61_BM:3 wt.% Sb_2_S_3_ films on ITO substrates

The micromorphologies of the active layers were further investigated with AFM in tapping mode to reveal the effects of the addition of CdS or Sb_2_S_3_. The height images of a pristine P3HT:PC_61_BM film and two ternary films with 3 wt.% CdS and 3 wt.% Sb_2_S_3_ are shown in Fig. 6. The surface morphology of the P3HT:PC_61_BM:3 wt.% CdS and P3HT:PC_61_BM:3 wt.% Sb_2_S_3_ layer show an obvious increase in surface roughness with the root mean square roughness increasing from 2.82 to 8.89 nm and 7.13 nm, respectively. The larger roughness observed for the P3HT:PC_61_BM:3 wt.% CdS and P3HT:PC_61_BM:3 wt.% Sb_2_S_3_ film could be a consequence of the presence of the CdS and Sb_2_S_3_ nanocrystals in the P3HT:PC_61_BM active layer. The CdS or Sb_2_S_3_ nanocrystals are thought to serve as a medium for enhancing the interpenetration of P3HT molecules and PC_61_BM in the composite film, leading to superior exciton dissociation. As a result of this superior exciton dissociation, the *J*_SC_ of the HSCs based on P3HT:PC_61_BM:3 wt.% CdS and P3HT:PC_61_BM:3 wt.% Sb_2_S_3_ were increased [51]. On the other hand, the incorporation of CdS or Sb_2_S_3_ nanocrystals in P3HT:PC_61_BM increases the surface roughness of the film, thus increases the interfacial contact area between the active layer (P3HT:PC_61_BM:3 wt.% CdS or P3HT:PC_61_BM:3 wt.% Sb_2_S_3_) and the hole transporting layer (MoO_3_). In this way, a more efficient hole collection at the anode was appeared, which might result in the improved *J*_SC_ and FF of the devices [52].

## Conclusions

In conclusion, as a doped material for offering superior charge mobility and enhancing light absorption, CdS or Sb_2_S_3_ nanocrystals were in situ generated inside the P3HT:PC_61_BM system by randomly mixing P3HT and PC_61_BM in the presence of cadmium or antimony xanthate precursor. The thermal stability of cadmium or antimony xanthate precursor and structure of the CdS or Sb_2_S_3_ films were characterized. The HSCs with the configuration of ITO/CdS interface layer/P3HT:PC_61_BM: x wt.% CdS/MoO_3_/Ag and ITO/CdS interface layer /P3HT:PC_61_BM: x wt.% Sb_2_S_3_/MoO_3_/Ag were fabricated. The effects of x wt.% CdS (or Sb_2_S_3_) nanocrystals on the performance of P3HT:PC_61_BM-based HSCs were studied. It has been proved that incorporation of CdS (or Sb_2_S_3_) nanocrystals in the active layer of P3HT:PC_61_BM-based solar cells helps in improving PCEs. And the highest PCEs of 2.91 and 2.92% were obtained for the HSCs with 3 wt.% CdS nanocrystals and 3 wt.% Sb_2_S_3_ nanocrystals, respectively. From UV–Vis absorption, hole mobilities, and surface morphological characterizations, our studies have suggested that 3 wt.% CdS or Sb_2_S_3_ embedded in P3HT:PC_61_BM matrix improved the optical absorption, the hole mobility and surface roughness in comparison with P3HT:PC_61_BM, thus resulting in the improved PCEs of the devices. The method of in situ generation of inorganic semiconductor nanocrystals inside the organic materials can be applied to design high-efficiency HSCs.

## Additional file

Additional file 1: **Figure S1.** SEM images of (a) ITO, (b) CdS thin films on ITO, and (c) Sb_2_S_3_ thin films on ITO. Figure S2 SEM images of (a) P3HT:PC_61_BM, (b) P3HT:PC_61_BM:3 wt.% CdS, and (c) P3HT:PC_61_BM:3 wt.% Sb_2_S_3_ films on ITO substrates. (ZIP 3399 kb)
