# Supplementary material for: In Situ Growth of Metal Sulfide Nanocrystals in Poly(3-hexylthiophene): [6,6]-Phenyl C61-Butyric Acid Methyl Ester Films for Inverted Hybrid Solar Cells with Enhanced Photocurrent
Source: Nanoscale Res Lett. 2018 Jun 20;13:184. doi: 10.1186/s11671-018-2596-0 (PMC6010366; doi:10.1186/s11671-018-2596-0)
Supplement: Supplementary file 1 — Figure S1. SEM images of (a) ITO, (b) CdS thin films on ITO, and (c) Sb2S3 thin films on ITO. Figure S2 SEM images of (a) P3HT:PC61BM, (b) P3HT:PC61BM:3 wt.% CdS, and (c) P3HT:PC61BM:3 wt.% Sb2S3 films on ITO substrates. (ZIP 3399 kb) [file 11671_2018_2596_MOESM1_ESM.zip › Supplementary Data.docx]

**Supplementary Data**

In situ growth of metal sulfide nanocrystals in poly(3-hexylthiophene): [6,6]-phenyl C61-butyric acid methyl ester films for inverted hybrid solar cells with enhanced photocurrent

Chunyan Yang,^*a^ Yingying Sun,^a^ Xinjie Li,^b^ Cheng Li,^a^ Junfeng Tong,^a^ Jianfeng Li,^a^ Peng Zhang,^a^ Yangjun Xia^a^


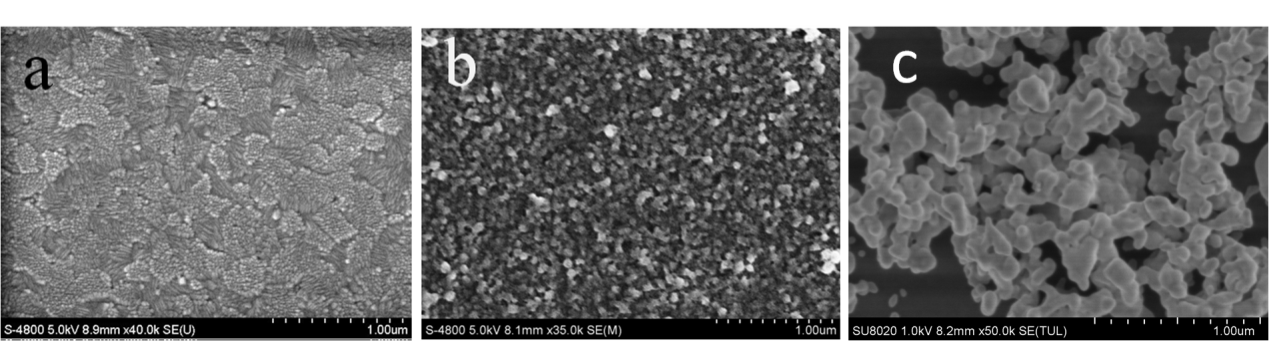


**Fig. S1.** SEM images of (a) ITO, (b) CdS thin films on ITO and (c) Sb_2_S_3_ thin films on ITO.


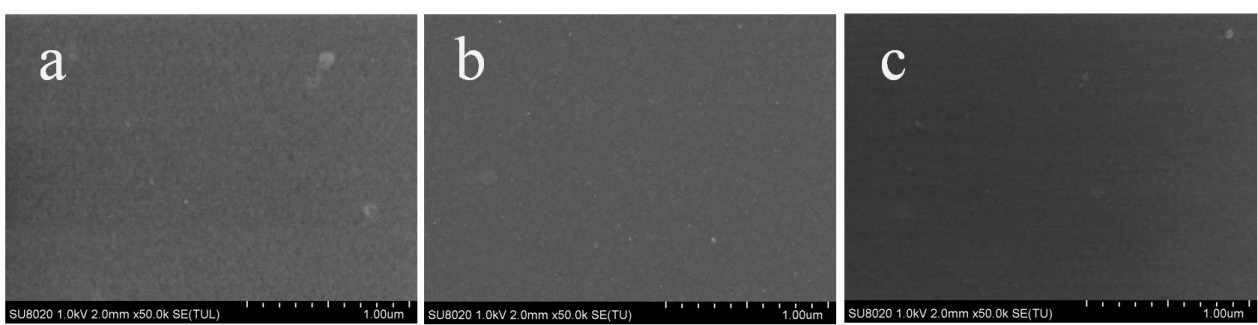


**Fig. S2.** SEM images of a) P3HT:PC_61_BM, (b) P3HT:PC_61_BM:3 wt.% CdS and (c) P3HT:PC_61_BM:3 wt.% Sb_2_S_3_ films on ITO substrates

.
