# Supplementary figures and images for: In Situ Growth of Metal Sulfide Nanocrystals in Poly(3-hexylthiophene): [6,6]-Phenyl C61-Butyric Acid Methyl Ester Films for Inverted Hybrid Solar Cells with Enhanced Photocurrent
Source: Nanoscale Res Lett. 2018 Jun 20;13:184. doi: 10.1186/s11671-018-2596-0 (PMC6010366; doi:10.1186/s11671-018-2596-0)

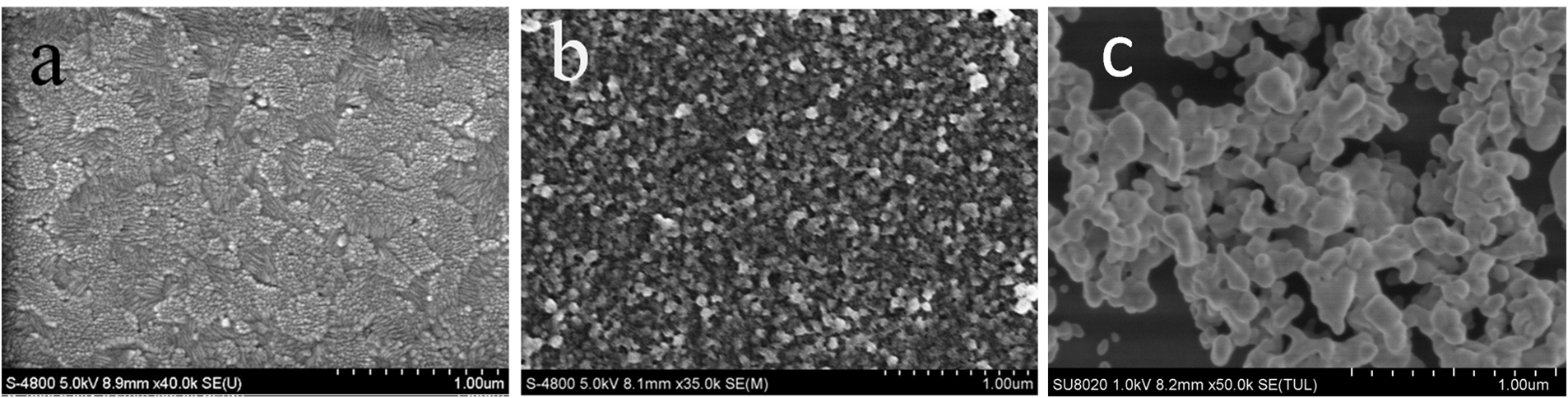

Supplement: Supplementary file 1 — Figure S1. SEM images of (a) ITO, (b) CdS thin films on ITO, and (c) Sb2S3 thin films on ITO. Figure S2 SEM images of (a) P3HT:PC61BM, (b) P3HT:PC61BM:3 wt.% CdS, and (c) P3HT:PC61BM:3 wt.% Sb2S3 films on ITO substrates. (ZIP 3399 kb) [file 11671_2018_2596_MOESM1_ESM.zip › Fig S1.tif]

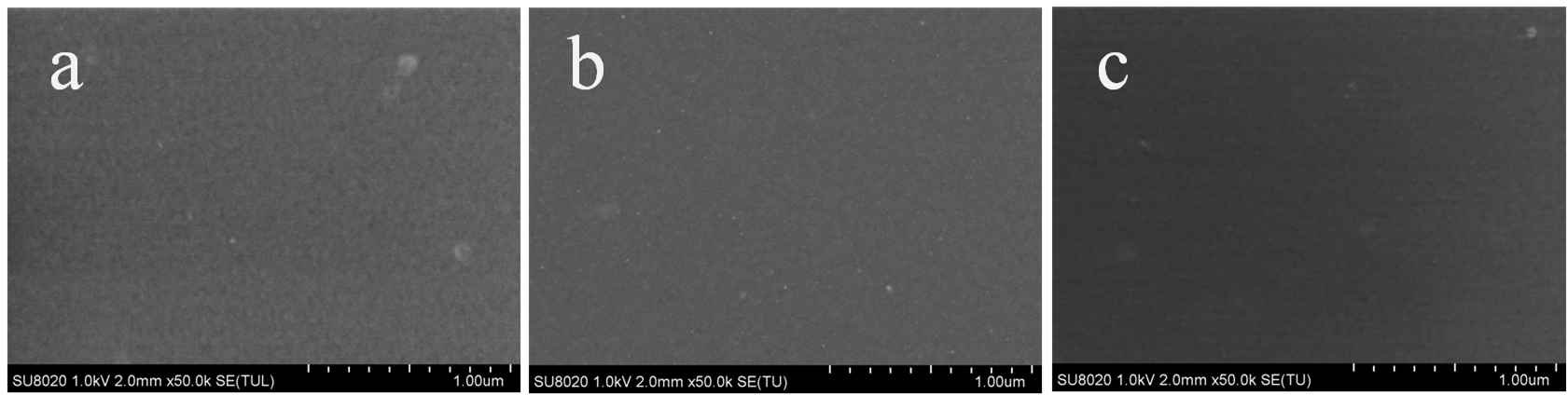

Supplement: Supplementary file 1 — Figure S1. SEM images of (a) ITO, (b) CdS thin films on ITO, and (c) Sb2S3 thin films on ITO. Figure S2 SEM images of (a) P3HT:PC61BM, (b) P3HT:PC61BM:3 wt.% CdS, and (c) P3HT:PC61BM:3 wt.% Sb2S3 films on ITO substrates. (ZIP 3399 kb) [file 11671_2018_2596_MOESM1_ESM.zip › Fig S2.tif]
